# Four Weekly Ayahuasca Sessions Lead to Increases in “Acceptance” Capacities: A Comparison Study With a Standard 8-Week Mindfulness Training Program

**DOI:** 10.3389/fphar.2018.00224

**Published:** 2018-03-20

**Authors:** Joaquim Soler, Matilde Elices, Elisabeth Dominguez-Clavé, Juan C. Pascual, Amanda Feilding, Mayte Navarro-Gil, Javier García-Campayo, Jordi Riba

**Affiliations:** ^1^Department of Psychiatry, Hospital de la Santa Creu i Sant Pau, Universitat Autònoma de Barcelona, Barcelona, Spain; ^2^Centro de Investigación Biomédica en Red de Salud Mental, Madrid, Spain; ^3^Department of Pharmacology, Therapeutics and Toxicology, Universitat Autònoma de Barcelona (UAB), Barcelona, Spain; ^4^The Beckley Foundation, Oxford, United Kingdom; ^5^Primary Care Prevention and Health Promotion Research Network, University of Zaragoza, Zaragoza, Spain; ^6^Miguel Servet Hospital, University of Zaragoza, Zaragoza, Spain; ^7^Human Neuropsychopharmacology Group, Sant Pau Institute of Biomedical Research, Barcelona, Spain

**Keywords:** ayahuasca, mindfulness, acceptance, Non-Judging, human

## Abstract

**Background:** The therapeutic effects of the Amazonian plant tea ayahuasca may relate to its ability to enhance mindfulness capacities. Ayahuasca induces a modified state of awareness through the combined action of its active principles: the psychedelic *N,N-*dimethyltryptamine (DMT) and a series of centrally acting β-carbolines, mainly harmine and tetrahydroharmine. To better understand the therapeutic potential of ayahuasca, here we compared the impact on mindfulness capacities induced by two independent interventions: (a) participation in four ayahuasca sessions without any specific purpose related to improving mindfulness capacities; and (b) participation in a standard mindfulness training course: 8 weeks mindfulness-based stress reduction (MBSR), with the specific goal of improving these skills.

**Methods:** Participants of two independent groups completed two self-report instruments: The Five Facet Mindfulness Questionnaire (FFMQ) and the Experiences Questionnaire (EQ). The MINDSENS Composite Index was also calculated, including those EQ and FFMQ items that have proven to be the most sensitive to meditation practice. Group A (*n* = 10) was assessed before and after the last of four closely spaced consecutive ayahuasca sessions. Group B (*n* = 10) was assessed before and after completion of a standard 8-week MBSR course.

**Results:** MBSR training led to greater increases in overall mindfulness scores after the 8-week period. MBSR but not ayahuasca led to increases in the MINDSENS Composite Index. However, the ayahuasca sessions induced comparable increases in the Non-Judging subscale of the FFMQ, specifically measuring “acceptance.” Improving this capacity allows for a more detached and less judgmental stance toward potentially distressing thoughts and emotions.

**Results:** The present findings suggest that a small number of ayahuasca sessions can be as effective at improving acceptance as more lengthy and costly interventions. Future studies should address the benefits of combining ayahuasca administration with mindfulness-based interventions. This will allow us to investigate if ayahuasca will improve the outcome of psychotherapeutic interventions.

## Introduction

In recent years there has been a renewed interest in the potential use of psychedelics for the treatment of different psychiatric conditions (Sessa, 2005; Mithoefer et al., 2016). One of the substances that have gained attention is ayahuasca; a tea obtained from the mix of *Banisteriopsis caapi* with *Psychotria viridis* (Rubiaceae) or *Diplopterys cabrerana* (Malpighiaceae) (McKenna et al., 1984). The β-carboline alkaloids present in ayahuasca [i.e., harmine, tetrahydroharmine (THH), and harmaline] show monoamine-oxidase (MAO) inhibiting properties (Buckholtz and Boggan, 1977b) and also serotonin reuptake inhibition (THH; Buckholtz and Boggan, 1977a). The leaves of *P. viridis* and *D. cabrerana* contain *N,N*-dimethyltryptamine (DMT), an alkaloid that is also extracted into the ayahuasca brew during the infusion process. DMT is the main psychotropic agent of ayahuasca, and possibly the responsible for the dream-like experience induced by the tea. This modified state of consciousness is characterized by the presence of visual imagery and the recollection of highly emotional autobiographic memories (Riba et al., 2001). On a molecular level, DMT has affinity for 5-HT_2A_ and 5-HT_1A_ binding sites, where it acts as an agonist or partial agonist (Riba et al., 2001; Carbonaro et al., 2015). Although DMT has been regarded as the primary ayahuasca compound acting on the CNS, recent research has shown that the β-carbolines may also have a relevant contribution to the overall effects of ayahuasca in the brain. Specifically, harmine, THH, and the harmine metabolite harmol, stimulate adult neurogenesis *in vitro* (Morales-García et al., 2017). Traditionally, ayahuasca has been consumed for ritual and medical purposes in the Amazon Basin. Today its use has spread worldwide, encouraging research on its potential therapeutic effects (Domínguez-Clavé et al., 2016). Compared to non-users, habitual ayahuasca consumers show lower hopelessness (Santos et al., 2007) and depression levels, and higher scores on certain personality traits like agreeableness and openness (Barbosa et al., 2016). Experimental studies of acute ayahuasca administration to healthy volunteers have found that ayahuasca targets key nodes of the default mode network (Valle et al., 2016; Sampedro et al., 2017) that are associated with higher self-consciousness and pathological ruminations (Vogt and Laureys, 2005). Additionally, data shows increased blood flow in several brain regions implicated in cognitive control, emotion regulation, and memory (Riba et al., 2006; Sanches et al., 2016). Recent clinical studies on its utility as an adjunct to psychological interventions have demonstrated therapeutic benefits in treatment-resistant depression (Osório Fde et al., 2015; Dos Santos et al., 2016; Sanches et al., 2016) and substance abuse (Fábregas et al., 2010; Thomas et al., 2013).

In a previous work by our group (Soler et al., 2016), we argued that the therapeutic effects of ayahuasca might be related to increases in mindfulness-related capacities. Mindfulness entails a focus on the present experience and reaching a state of non-judgmental awareness, enhanced curiosity and openness (Kabat-Zinn, 1990; Bishop et al., 2004; Baer et al., 2006). These qualities can be considered from a dimensional trait perspective, but can also be fostered through meditative practice. In the last three decades there has been a proliferation of mindfulness-based interventions designed to teach individuals how to maximize these skills. Probably the most commonly used intervention is the Mindfulness-Based Stress Reduction (MBSR; Kabat-Zinn, 1990) approach, an 8-week program that has been widely applied to deal with a number of medical and psychiatric conditions. MBSR focuses on the cultivation of mindfulness through formal meditation practices (i.e., body scan, sitting meditation and yoga), and on the integration of mindfulness-principles into everyday activities (Kabat-Zinn, 1990). In our previous study (Soler et al., 2016), we assessed mindfulness-related capacities before and after one dose of ayahuasca, finding that ayahuasca intake led to increases in three core mindfulness facets: decentering, defined as the capacity to observe one’s thoughts and inner experiences in a detached manner (Fresco et al., 2007); Non-Judging and Non-Reacting, defined, respectively, as the ability to take non-judgmental and non-reactive stances toward emotions, thoughts and experiences in general (Baer et al., 2006). Moreover, in a subsequent study using magnetic resonance imaging (MRI) we reported that post-acute metabolic and connectivity changes in the brain after a single ayahuasca session were associated with maintained elevations in the non-judgmental attitudes 2 months later (Sampedro et al., 2017).

Together, the above data indicates that traditional mindfulness training techniques are not the only pathway to foster mindfulness capacities. They further suggest that ayahuasca intake may attain analogous results (Soler et al., 2016). However, the specific domains targeted by either approach have not been assessed. Here, we conducted an exploratory-comparison study in order to evaluate the similarities and differences of the two approaches. Specifically, we compared the impact on mindfulness scores of: (a) participation in four consecutive ayahuasca sessions without the specific purpose of improving mindfulness capacities; and (b) participation in a standard mindfulness training course (8 weeks MBSR), with the specific goal of improving these skills. We hypothesized that both interventions would result in significant improvements in mindfulness capacities. However, given the lack of previous research comparing these two interventions hypotheses in regards to changes in specific-mindfulness facets were not made.

## Materials and Methods

### Participants

A total of 20 individuals (ten per group) were enrolled in the study. Groups were comparable in terms of age [ayahuasca group mean age = 50.00 years (*SD* = 14.71); MBSR group mean age = 42.00 years (*SD* = 11.44; *F* = 1.84, *p* = 0.19)] and sex (seven females in each group). Based on a previous study (Soler et al., 2016) where ayahuasca users demonstrated unusually high baseline scores on decentering, as measured by the Experiences Questionnaire (EQ), we used the single-factor EQ scale to match participants between groups. **Table 1** shows that there were not baseline differences between ayahuasca and MBSR groups in neither FFMQ facets or the EQ.

**Table 1:** Differences in FFMQ and EQ baseline scores between participants in the ayahuasca group and participants in the mindfulness group.

	Ayahuasca (*n* = 10)		MBSR (*n* = 10)			
	*M*	*SD*	*M*	*SD*	*F*	*p*
**FFMQ**						
Observing	25.70	8.08	25.10	1.72	0.05	0.82
Describing	29.00	9.36	26.50	2.01	0.68	0.42
Acting with awareness	30.70	6.36	26.90	0.87	3.50	0.80
Non-Judging	30.00	9.32	27.10	0.73	0.96	0.34
Non-Reacting	22.00	8.39	23.40	1.77	0.26	0.61
**EQ**	38.00	9.62	39.00	3.49	0.09	0.76

*MBSR, Mindfulness-Based Stress Reduction; M, Mean; SD, Standard Deviation; FFMQ, Five Facets Mindfulness Questionnaire; EQ, Experiences Questionnaire*.

Individuals interested in participating in more than one ayahuasca session were contacted and received information about the study aims. They were also asked to pass the information to their acquaintances. The participant’s principal motivation was to use ayahuasca to increase self-knowledge and introspection. To be included in the study, participants in the ayahuasca group needed to: (1) have abstained from ayahuasca, medications or illicit drugs at least 2 weeks before the initial assessment, (2) have abstained from alcohol, in the 24 h prior to the initial and final assessments; and (3) agree to abstain from taking any drug other than ayahuasca for the entire duration of the study. To increase recruitment success, prior experience with ayahuasca was not an exclusion criterion. No participant in the ayahuasca group reported having any meditation experience.

The mindfulness group consisted of individuals naïve to meditation who were interested in mindfulness and who had enrolled in a MBSR course. The study’s aims were explained to them before the beginning of the course. Inclusion criteria for the MBSR group were: (1) no history of ayahuasca consumption; (2) no alcohol intake in the 24 h prior to the initial and final assessments; and (3) agree to abstain from taking any psychoactive drug for the entire duration of the study.

The study was conducted in accordance with the Declaration of Helsinki and was approved by the Sant Pau Hospital Ethics Committee. All participants gave their written informed consent prior to participation.

### Study Groups

#### Ayahuasca Group

Participants were recruited from a pool of individuals who had freely decided to participate in ayahuasca sessions in the Barcelona area, in a non-religious setting. Ayahuasca was taken in a dimly light room with participants sitting or lying down on mattresses while recorded music was played. Participants were free to leave the room if they so desired. Experimenters were present before, during, and after each session, sessions lasted between 6 and 8 h. Questionnaires were administered before the first and 24 h after the last of four consecutive sessions held a week apart.

#### MBSR Group

Participants in this group participated in a standard MBSR program consisting of weekly 2.5 h sessions for eight consecutive weeks. Training was conducted according to the MBSR guidelines (Kabat-Zinn, 1990). During the 8 weeks participants were trained in three types of mindfulness techniques. Through “body scan,” participants learn to focus attention sequentially on parts of the body, non-judgmentally noticing any sensation that might be present. The second technique involves practicing mindful hatha yoga postures to develop body awareness through gentle movements and stretching. The third, “sitting meditation” involves using awareness of the sensations associated with breathing as an anchor, while noticing other bodily sensations, sounds or thoughts. In the course of the program participants were also encouraged to conduct informal mindfulness exercises, by carrying out everyday activities (e.g., eating, walking, washing the dishes) with a full awareness of the movements, sensations, cognitions, and feelings that may be involved in each particular task. CD’s containing recorded formal meditation practices were given to participants, who were encouraged to practice at home by listening to the CD 45 min each day throughout the duration of the program (Kabat-Zinn, 1990). Between sessions 5 and 6, individuals participated in a “day of silence” during which they were guided through various practices. Participants in the MBSR group filled out the administered questionnaires 24 h prior to the first MBSR session and after the eighth and final MBSR session.

### Measures

To assess mindfulness-related capacities the Spanish versions of two instruments were used.

The *Five Facet Mindfulness Questionnaire or FFMQ* (Baer et al., 2006; Cebolla et al., 2012) is a self-reported questionnaire that measures five mindfulness components: (1) Observing: noticing external and internal experiences, e.g., body sensations, thoughts or emotions; (2) Describing: putting words to, or labeling the internal experience; (3) Acting with awareness: focusing on the present activity instead of behaving mechanically; (4) Non-Judging the inner experience: taking a non-evaluative stance toward the present experience, thoughts or emotions; and (5) Non-Reacting to the inner experience: allowing thoughts and feelings to come, without getting caught up in, or carried away, by them. Sample items for each sub-scale include: Observing “When I take a shower or bath, I stay alert to the sensations of water on my body”; Describing “I’m good at finding words to describe my feelings”; Acting with awareness “I am easily distracted”; Non-Judging “I tell myself I should not be feeling the way I am feeling”; and Non-Reacting “I watch my feelings without getting lost in them.” Study participants were asked to rate the degree of agreement with each statement on a 5-point scale that ranges from 1 (*never, or very rarely true*) to 5 (*very often, or always, true*). After reverse scoring specific items, mean ratings are calculated for each of the five facets. Facet scores range from 8 to 40, with the exception of the non-reactivity facet, which ranges from 7 to 35 and higher scores reflect greater mindfulness.

The “Non-Judging” and “Non-Reacting” factors represent the “acceptance” component of the FFMQ, while the other factors are more related to the attentional aspect of mindfulness (Baer et al., 2006).

The *EQ* (Fresco et al., 2007; Soler et al., 2014b) was used as a measure of decentering. The EQ has 11 items and measures a metacognitive ability known as “decentering,” i.e., the capacity to observe one’s thoughts and emotions in a detached manner, considering them transient events of the mind. Sample items include: “I can observe unpleasant feelings without being drawn into them” or “I can separate myself from my thoughts and feelings.” The EQ items are scored in a 5-point scale, ranging from *never* to *all the time*, with higher scores indicating more decentering. EQ scores are obtained by adding the scores of each item and dividing them by the total number of items.

The MINDSENS Composite Index was also calculated (Soler et al., 2014a). This index includes those EQ and FFMQ items that have proven to be the most sensitive to meditation practice (Soler et al., 2014a). The MINDSENS Composite Index is the average of the sum of 9 items of the EQ and 10 items of the FFMQ (corresponding to the observing and non-reacting sub-scales; Soler et al., 2014a).

### Data Analysis

Between groups differences at pre-intervention in mindfulness scores (FFMQ and EQ) were explored by means of an ANOVA. FFMQ, EQ, and MINDSENS scores were analyzed by means of multivariate (FFMQ subscale scores) and univariate (EQ and MINDSENS scores) repeated-measures analyses of variance (ANOVAs). FFMQ subscale scores, EQ scores and MINDSENS scores were entered in the respective ANOVAs as the dependent variables. Participant group (ayahuasca vs. mindfulness) was entered as a between-subjects factor and time (pre- and post-assessment) as a within subject’s factor. *Post hoc* analyses were conducted using Student’s *t*-test. Results were considered significant for *p*-values < 0.05.

## Results

The multivariate-repeated measures ANOVA using FFMQ’s scores as the dependent variables showed a significant effect of time × group [*F*(5,14) = 5.16, *p* = 0.007]. Univariate tests showed a significant time × group effect for the following FFMQ facets: Observing [*F*(1,19) = 22.60, *p* < 0.001], Describing [*F*(1,19) = 13.61, *p* = 0.00], Acting with awareness [*F*(1,19) = 8.52, *p* = 0.01], and Non-Reacting [*F*(1,19) = 6.50, *p* = 0.02]. For scores on Non-Judging no significant time × group effect was found [*F*(1,19) = 3.43, *p* = 0.08]. Participants in the mindfulness group showed significant pre–post treatment increases in all mindfulness facets: Observing [*t*(9) = -17.14, *p* < 0.001], Describing [*t*(9) = -9.18, *p* < 0.001], Acting with awareness [*t*(9) = -18.46, *p* < 0.001], Non-judging [*t*(9) = -14.95, *p* < 0.001], and Non-Reacting [*t*(9) = -8.15, *p* < 0.001]. In the ayahuasca group a significant pre–post improvement was found for Non-Judging [*t*(9) = -2.67, *p* = 0.02], while no significant pre–post differences were observed for the remaining FFMQ facets: Observing [*t*(9) = -1.10, *p* = 0.29], Describing [*t*(9) = -1.45, *p* = 0.17], Acting with awareness [*t*(9) = -1.86, *p* = 0.09], and Non-Reacting [*t*(9) = -1.23, *p* = 0.25].

For EQ scores a significant time × group interaction was found [*F*(1,18) = 7.87, *p* = 0.01] and *post hoc* analyses showed significant pre–post differences in the mindfulness group [*t*(9) = -9.63, *p* < 0.001], whereas no significant differences were found for the ayahuasca group [*t*(9) = -1.82, *p* = 0.10].

**FIGURE 1:**
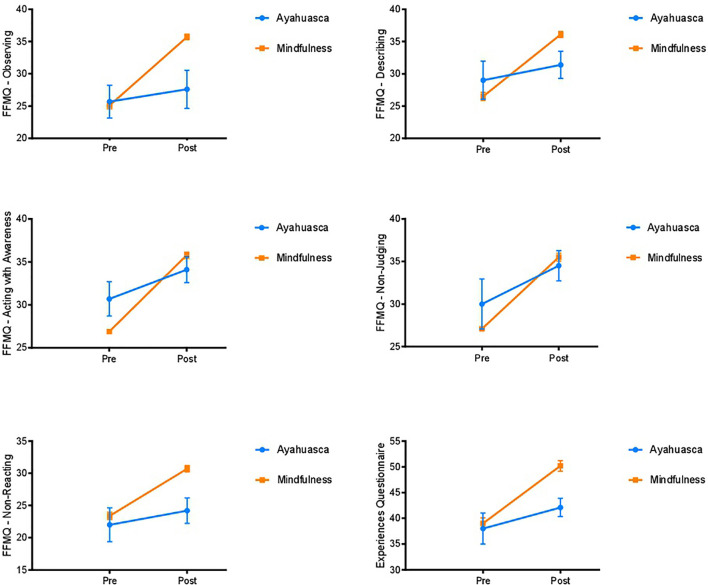
Pre–post changes in FFMQ facets and decentering scores (Experiences Questionnaire) in group A (ayahuasca intake) and group B (mindfulness training). Values are shown as means with standard errors represented by vertical bars.

The ANOVA for the MINDSENS Composite Index also revealed a significant time × group effect [*F*(1,18) = 21.13, *p* < 0.001]. *Post hoc* analyses showed significant differences in the mindfulness group [*t*(9) = -17.84, *p* < 0.001], but not in the ayahuasca group [*t*(9) = -1.50, *p* = 0.17]. **Table 2** and **Figure 1** show pre–post mindfulness scores for each group.

**Table 2:** Comparison of FFMQ and EQ scores between participants in the ayahuasca group and participants in the mindfulness group.

	Ayahuasca (*n* = 10)				MBSR (*n* = 10)					
	Pre		Post		Pre		Post		Group × time	
	*M*	*SD*	*M*	*SD*	*M*	*SD*	*M*	*SD*	*F*	*p*
**FFMQ**						
Observing	25.70	8.08	27.60	9.38	25.10	1.72	35.70**	1.33	22.60	<0.001
Describing	29.00	9.36	31.40	6.73	26.50	2.01	36.10**	1.52	13.61	0.00
Acting with awareness	30.70	6.36	34.10	4.81	26.90	0.87	35.80**	1.13	8.52	0.01
Non-Judging	30.00	9.32	34.50*	5.66	27.10	0.73	35.50**	1.43	3.43	0.08
Non-Reacting	22.00	8.39	24.20	6.30	23.40	1.77	30.70**	1.63	6.50	0.02
**EQ**	38.00	9.62	42.10	5.66	39.00	3.49	50.20**	3.35	7.87	0.01
**MINDSENS**	3.33	0.91	3.61	0.74	3.31	0.16	4.50**	0.19	21.13	<0.001

*MBSR, Mindfulness-Based Stress Reduction; M, Mean; SD, Standard Deviation; FFMQ, Five Facets Mindfulness Questionnaire; EQ, Experiences Questionnaire ^∗^*p* < 0.05; ^∗∗^*p* < 0.001*.

## Discussion

The current study aimed to better understand the psychological mechanisms related to ayahuasca’s therapeutic effects by studying its impact on mindfulness-related capacities. Extending our previous work (Soler et al., 2016), here we compared the effects of four consecutive ayahuasca sessions with those of a standard mindfulness training. Whereas individuals in the ayahuasca group had decided to take the psychedelic for personal reasons unrelated to the objectives of our study, those receiving standard mindfulness training (i.e., MBSR), had enrolled in the program with the specific purpose of improving their mindfulness capacities. Results showed that mindfulness training had an overall greater impact on mindfulness capacities. However, ayahuasca intake led to spontaneous increases in certain aspects of the “acceptance” domain, as measured by the Non-Judging subscale of the FFMQ. Acceptance is one the psychological spheres cultivated in mindfulness meditation and in mindfulness-based psychotherapy. Increasing acceptance enables the individual to attain a non-evaluative stance toward their experience of “being,” their thoughts and their emotions without getting carried away by them, no matter how painful these may be.

Our results concerning the Non-Judging subscale replicate the findings obtained by our group in two prior independent studies that assessed ayahuasca users after a single session (Soler et al., 2016; Sampedro et al., 2017). In both cases, ayahuasca increased the scores on the Non-Judging and Non-Reacting subscales of the FFMQ, which focus on the “acceptance” domain of mindfulness abilities. Similar to our present results, these previous studies did not find any effect of ayahuasca on the other three subscales of the FFMQ that assess the “attention” domain of mindfulness abilities. Interestingly, in the Sampedro et al. (2017) study a follow-up assessment was carried out 2 months after the ayahuasca session and found that Non-Judging was the only subscale that remained elevated (Sampedro et al., 2017). The present findings thus further support for the following ideas: (a) ayahuasca intake leads to psychological modifications that are observable beyond the time frame of the acute inebriation; and (b) a reduction in self-judgmental patterns of thought is a key feature of the post-acute stage.

A novel and relevant finding in the present investigation is that both ayahuasca and MBSR produce improvement in acceptance, although only the latter -MBSR- is a theoretical and practical training program specifically designed to foster mindfulness domains (Gu et al., 2015; Khoury et al., 2015). While individuals in the ayahuasca group may have had a wide variety of reasons to engage in ayahuasca taking, subjects in the comparison group had enrolled in the MBSR course with the explicit purpose of enhancing mindfulness capacities. Importantly, ayahuasca would thus appear to have the potential to increase acceptance *per se*, without this being a manifest and desired outcome of ayahuasca intake. Although studies with larger sample size are needed, the above finding is relevant for a number of reasons, theoretical and clinical. First, reducing automatic judgmental attitudes is particularly hard to achieve. Montero-Marin et al. (2016) found improvements in Observing, Non-Reacting, decentering and non-attachment in individuals who had participated in a 1-month meditation retreat, but failed to find modifications in Non-Judging. In another study, the authors assessed the influence of practice (i.e., frequency of meditation, session duration, lifetime practice) on various mindfulness facets. Results showed that Non-Judging improved with practice significantly less than Non-Reacting, Observing and decentering (Soler et al., 2014a). Second, acceptance has been found to play a fundamental role in psychological health. Non-judgmental awareness is commonly impaired in diverse clinical populations (Coffey et al., 2010). Deficits have been reported for individuals with eating disorders (Lavender et al., 2011), borderline personality disorder (Baer et al., 2004), and cocaine use disorder (Tejedor et al., 2014). Moreover, Brown et al. (2015) have found an association between higher Non-Judging capacities and lower depressive symptoms and anxiety. Kotsou et al. (2018), showed that higher acceptance is a better predictor of lower psychopathology than other variables related to mental health such as emotional competence, emotion regulation or even present-centered awareness. Interestingly, increasing acceptance has been directly linked to the positive outcomes of mindfulness practice (Holzel et al., 2011), and of exposure interventions (Brown et al., 2015). The acute stage of ayahuasca has been assimilated to a controlled exposure to autobiographical material (Domínguez-Clavé et al., 2016). Our results suggest that acute exposure is followed by a subsequent stage of increased acceptance. These combined effects could be of great value in a psychotherapeutic context (Domínguez-Clavé et al., 2016; Mithoefer et al., 2016).

From a neurobiological perspective, our findings can be linked to the selective modulation by ayahuasca of specific brain regions and networks. In a neuroimaging study using MRI, we found associations between Non-Judging increases and post-acute neurometabolic and connectivity changes (Sampedro et al., 2017). Reductions in the levels of the excitatory glutamate–glutamine complex in the posterior cingulate cortex (PCC) correlated with Non-Judging increases 24 h after ayahuasca intake and at follow-up 2 months later. In the same study, increased functional connectivity between the anterior cingulate cortex (ACC) and the PCC and between the ACC and the medial temporal lobe (MTL) also correlated with Non-Judging at the two assessment time points. Thus, desirable effects at the psychological level were linked on the one hand to neurometabolic reductions in the PCC, a region which is key to the sense of self (Vogt and Laureys, 2005) and is abnormally hyperactive in certain psychiatric conditions (Hamilton et al., 2011). On the other hand, enhanced Non-Judging relied also on increased neural network cross-talk and on an increased coupling of activity between the ACC, a key center of self-monitoring and other aspects of executive function (Kelly et al., 2009) and the MTL, a limbic region processing memory and emotion (Dolcos et al., 2004).

Contrary to our previous findings (Soler et al., 2016; Sampedro et al., 2017), scores on the Non-Reacting subscale of the FFMQ and on the EQ questionnaire (decentering) were not significantly modified after ayahuasca. These discrepancies may indicate a less robust effect of ayahuasca on these variables. As a matter of fact, in our previous neuroimaging study we found that post-acute increases in these variables had returned to baseline levels 2 months later, while scores on Non-Judging remained elevated (Sampedro et al., 2017). Another possible explanation could be that Non-Judging is difficult to increase and thus less prone to show ceiling effects or erratic behavior (see limitations paragraph below). The fact that ayahuasca users were only assessed twice, i.e., before and after fourth ayahuasca session may have prevented us from detecting these potential problems. This brings up the question of the potential impact of the pattern of ayahuasca consumption (e.g., frequency, amount, time between intakes) on the modulation of each mindfulness facet and domain. This question warrants further research in order to optimize the number of ayahuasca sessions and the spacing between sessions in future therapeutic studies.

The observational and exploratory nature of our study involved several limitations that need to be mentioned. The main limitation of the present study was the small sample size that limited the statistical power of the study. Assessments were only conducted, respectively, before the first and after the fourth ayahuasca session, and before and at the end of the MBSR course. No assessments were conducted in between. This approach was selected to avoid imposing an excessive burden on the ayahuasca-using participants that might have led to high drop-out levels. The fact that individuals in the ayahuasca group had prior experience with this psychedelic may have led to ceiling effects on the study variables. On the other hand, the higher scores obtained for several variables in the MBSR group may have been biased by the fact that participants in the mindfulness training course had the explicit intention of enhancing their mindfulness capacities. Ideally, future studies could assign ayahuasca-naïve and meditation-nature participants to either group randomly. In order to shorten the assessment, we did not explore subjective effects of ayahuasca intake nor the specific motivations to attend to ayahuasca sessions. In addition, including a placebo control group for the ayahuasca condition could also have been interesting to assess possible placebo effects. Assessments could be conducted in both groups at several time points along the study period and follow-up data gathered in order to determine the temporal stability of the findings. Additionally, the design of the study did not include alkaloid determinations in the ayahuasca used by the participants. Lastly, and although participants did not report any current psychiatric disorder or medical condition, no formal medical history was obtained.

## Conclusion

To conclude, the present results suggest that the “acceptance” domain of mindfulness capacities is particularly sensitive to improvement by ayahuasca, and potentially other psychedelics. Together with previous findings (Soler et al., 2016; Sampedro et al., 2017), the current results open the interesting possibility of using ayahuasca as a tool to enhance acceptance in the context of psychotherapy. Our findings indicate that a small number of ayahuasca sessions could be effective at improving acceptance, similarly to more lengthy and costly interventions. The present findings should be interpreted in light of the aforementioned limitations. The small sample size compromised the statistical power of the study, increasing the possibility of false positive and false negatives. Studies with a larger sample size are needed to confirm the findings of this exploratory study. Future studies should address the benefits of combining ayahuasca administration with mindfulness-based interventions, in order to investigate if ayahuasca will improve the outcome of these psychotherapeutic interventions.

## Author Contributions

JS, JP, and JR conceived the study. ME and ED-C performed the statistical analyses and drafted the first version of the manuscript. JG-C and MN-G performed the mindfulness intervention. AF contributed to the interpretation of the study’s findings. All authors contributed to the writing and reviewing of the manuscript.

## Conflict of Interest Statement

The authors declare that the research was conducted in the absence of any commercial or financial relationships that could be construed as a potential conflict of interest.
