# Corrigendum: Four Weekly Ayahuasca Sessions Lead to Increases in “Acceptance” Capacities: A Comparison Study with a Standard 8-Week Mindfulness Training Program

**DOI:** 10.3389/fphar.2020.635111

**Published:** 2021-01-21

**Authors:** Joaquim Soler, Matilde Elices, Elisabeth Dominguez-Clavé, Juan C. Pascual, Amanda Feilding, Mayte Navarro-Gil, Javier Garcia-Campayo, Jordi Riba

**Affiliations:** ^1^ Department of Psychiatry, Hospital de la Santa Creu i Sant Pau, Universitat Autònoma de Barcelona, Barcelona, Spain; ^2^ Centro de Investigación Biomédica en Red de Salud Mental, Madrid, Spain; ^3^ Department of Pharmacology, Therapeutics and Toxicology, Universitat Autònoma de Barcelona (UAB), Barcelona, Spain; ^4^ The Beckley Foundation, Oxford, United Kingdom; ^5^ Primary Care Prevention and Health Promotion Research Network, University of Zaragoza, Zaragoza, Spain; ^6^ Miguel Servet Hospital, University of Zaragoza, Zaragoza, Spain; ^7^ Human Neuropsychopharmacology Group, Sant Pau Institute of Biomedical Research, Barcelona, Spain

**Keywords:** ayahuasca, mindfulness, acceptance, non-judging, human

In the published article, there was an error in affiliation 3. Instead of “Department of Psychiatry and Legal Medicine, Universitat Autònoma de Barcelona, Barcelona, Spain”, it should be “Department of Pharmacology, Therapeutics and Toxicology, Universitat Autònoma de Barcelona (UAB), Barcelona, Spain”.

In the published article, there was an error regarding the affiliations for Elisabet Dominguez-Clavé. As well as having affiliation 1, they should also have “Centro de Investigación Biomédica en Red de Salud Mental, Madrid, Spain” and “Department of Pharmacology, Therapeutics and Toxicology, Universitat Autònoma de Barcelona (UAB), Barcelona, Spain”. The authors apologize for this error and state that this does not change the scientific conclusions of the article in any way. The original article has been updated.

